# Internal validation of modified Mirels’ scoring system for pathologic femur fractures

**DOI:** 10.1186/s12891-024-07836-w

**Published:** 2024-09-06

**Authors:** Vishal S. Desai, Richard L. Amendola, Kenneth A. Mann, Timothy A. Damron

**Affiliations:** Department of Orthopedic Surgery, SUNY Upstate Medical University, 750 East Adams Street, Syracuse, NY 13210 USA

**Keywords:** Mirels scoring, Proximal femur, Pathologic fracture, Finite element, Decision curve analysis

## Abstract

**Background:**

The proximal femur is a common site of bone metastasis. The Mirels’ score is a frequently utilized system to identify patients at risk for pathologic fracture and while it has consistently demonstrated strong sensitivity, specificity has been relatively poor. Our group previously developed a Modified Mirels’ scoring system which demonstrated improved ability to predict cases at risk of fracture in this patient population through modification of the Mirels’ location score. The purpose of the present study is to internally validate this newly developed scoring system on an independent patient series.

**Methods:**

Retrospective review was performed to identify patients who were evaluated for proximal femoral bone lesions. Patients were stratified into one of two groups: 1) those who went on to fracture within 4 months after initial evaluation (Fracture Group) and 2) those who did not fracture within 4 months of initial evaluation (No Fracture Group). Retrospective chart review was performed to assign an Original Mirels’ (OM) Score and Modified Mirels’ (MM) score to each patient at the time of initial evaluation. Descriptive statistics, logistic regression, receiver operating curve, and net benefit analyses were performed to determine the predictability of fractures when utilizing both scoring systems.

**Results:**

The use of the MM scoring improved fracture prediction over OM scoring for patients observed over a 4 month follow up based on logistic regression. Decision curve analysis showed that there was a net benefit using the MM score over the OM scoring for a full range of fracture threshold probabilities. Fracture prevalence was similar for current internal validation dataset when compared to the dataset of our index study with a comparable reduction in misclassification of fracture prediction when utilizing the modified scoring system versus the original.

**Conclusions:**

Use of MM scoring was found to improve fracture prediction over OM scoring when tested on an internal validation set of patients with disseminated metastatic lesions to the proximal femur. The improvement in fracture prediction demonstrated in the present study mirrored the results of our index study during which the MM system was developed.

## Introduction

The proximal femur is a common site of bone metastasis, and identifying patients at risk for impending pathological fracture remains a clinical challenge. Mirels’ score classification [18] is a frequently utilized system to identify patients at risk for pathologic fracture. The appeal of Mirels’ scoring is that it relies on simple scoring of four components (pain level, lesion type, size, and location). The Mirels’ score is easily calculated and does not require any advanced computational tools to estimate fracture risk. While the sensitivity of the scoring system is strong (71–100%), the specificity is poor (13–50%) [5]. Strict application of Mirels’ scoring for presumed impending pathological fracture can capture the vast majority of cases that would fracture without prophylactic stabilization, but may also subject patients without true impending fractures (false positives) to unnecessary surgery.

Numerous efforts have been made to improve on the accuracy of fracture prediction [2, 9, 14, 15, 22, 24, 26] including modification to the Original Mirels’ (OM) scoring system [3, 13, 21, 25]. The OM scoring system assigned higher Mirels’ location scores to lesions located in the intertrochanteric and neck regions of the femur compared to those in the subtrochanteric and diaphyseal regions. However, Mirels anatomic location scoring does not individually predicted fracture risk or improves the accuracy when included in the total Mirels score. Biomechanical studies have shown higher strains in the subtrochanteric and diaphyseal regions of the femur during activities of daily living [16, 23], suggesting that lesions located in those regions might be at higher risk of fracture. Detailed finite element analyses of simulated lytic lesions at 32 unique locations of ten different proximal femurs subjected to gait and stair climbing loading also found that lesions located in the subtrochanteric and diaphyseal regions resulted in a greater risk of fracture [1]. Based on these findings, our group previously developed a Modified Mirels’ (MM) scoring system [1] which assigned higher location scores to subtrochanteric and diaphyseal lesion locations compared to those in the neck and intertrochanteric regions. A set of patients with disseminated tumors to the femur from an existing Musculoskeletal Tumor Society (MSTS) study [12] was used to test the accuracy of the MM scoring when compared to OM scoring. Application of MM scoring with a score ≥ 9 (indicating an impending pathologic fracture) resulted in a 20% improvement in specificity with no reduction is sensitivity. However, to date, no validation study has been performed.

The purpose of the present study was to internally validate this newly developed scoring system on a separate patient population with disseminated tumors to the femur using planar radiographic imaging. We asked three research questions: (1) Does MM scoring improve fracture prediction over OM scoring for patients observed over a 4 month follow up? (2) What is the net benefit of using MM scoring over OM scoring? (3) Are the findings from this validation study consistent with the results of the original MM study?

## Methods

### Study subjects

**Fig. 1:**
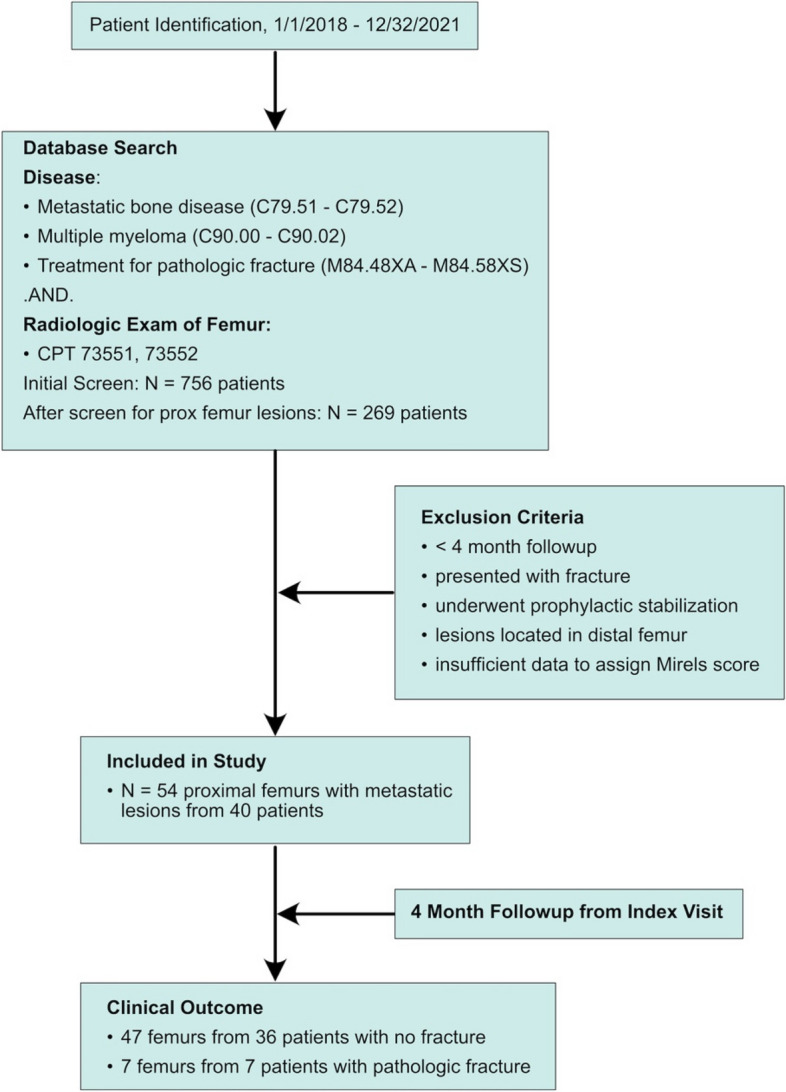
STROBE diagram. Legend: STROBE diagram to identify patients with impending pathological fracture and four-month follow-up

This study was approved by the Institutional Review Board (IRB) for the protection of human subjects from SUNY Upstate Medical University, Syracuse, NY (1787322–1) informed consent has been waived by the IRB. Also, the methods were in accordance with the 1964 Helsinki declaration and later amendments or comparable standards. Using the appropriate International Classification of Diseases Codes (ICD), patients who were clinically evaluated for metastatic disease to bone, multiple myeloma, or lymphoma between 1/1/2018 and 12/31/2021, as well as those who underwent treatment for pathologic fracture were identified within an institutional database (Fig. 1). Additionally, use of Current Procedural Terminology (CPT) codes denoting radiologic examination of the femur within the same time frame were also included in the initial search. The initial database search yielded 756 unique patients which was reviewed manually to identify 269 patients who were evaluated for proximal femoral bone lesions. Initial evaluation and follow-up were performed by the senior author (T.A.D.) for all patients.

Following removal of duplicate entries, chart review was performed to manually identify eligible patients and stratify them into one of two groups: 1) those who went on to fracture within 4 months after initial evaluation (*Fracture group*) and 2) those who did not fracture within 4 months after initial evaluation (*No Fracture group*). Patients were excluded if they had < 4 months of clinical follow-up from the time of initial evaluation, presented to our institution with a pathologic fracture, underwent prophylactic fixation, had a distal femur lesion (defined as being distal to the diaphyseal-metaphyseal junction), or insufficient clinical and radiographic data available to determine an accurate Original Mirels’ (OM) & Modified Mirels’ (MM) score. Eligible patients treated prior to January 1, 2018 were also excluded as they had been prospectively enrolled in our index MSTS study [1] used to evaluate the performance of MM scoring and thus ineligible for inclusion in a validation study. Forty patients ultimately met the criteria for inclusion in our study. Twenty-six patients had unilateral femoral lesions, while 14 had bilateral lesions. Of the 54 femurs that were initially evaluated for potential impending fracture, 7 went on to fracture and 47 did not during the 4 months observation period.

The 4-month follow-up window was chosen to replicate that used in the index study. This time period was initially chosen because fractures that might have occurred later (~ 1 year) would likely have progression of extent of metastatic lesions, but this would not have been represented in the initial imaging for the patient. Patients that presented with pathologic fractures were excluded because, 1) this population was not included in the index study, 2) there were uncertainties in assigning OM & MM scores to a patient after a fracture had already occurred, and 3) there is a lack of utility and rationale for scoring post-fracture patients for risk of fracture, when they had already fractured. Because the purpose of developing fracture risk prediction only applies to those who present clinically without a fracture, inclusion of patients who have already incurred a fracture would potentially introduce factors unique to that group that would not necessarily apply to those who have not yet fractured. They were therefore excluded to isolate a more clinically relevant, homogenous group. Exclusion of patients that underwent prophylactic stabilization could add bias to the study because it would not be possible to determine if these cases would have fractured if not treated.

The original independent evaluation dataset (referred to here as *MSTS test dataset*) was derived from patients at our institution previously enrolled in a Musculoskeletal Tumor Society Study (MSTS) study [1]. Briefly, eligibility criteria for the MSTS study included patients evaluated for disseminated metastatic carcinoma to the femur and were included regardless of prognosis or treatment. CT scans with hydroxyapatite (HA) phantom were obtained at the start of enrollment. Patients were followed for 4 months resulting in 8 fracture and 40 no fracture cases. The performance of the Mirels’ scoring systems (OM and MM) in the current validation study were compared to performance in the original MSTS dataset.

### Mirels’ scoring

**Fig. 2:**
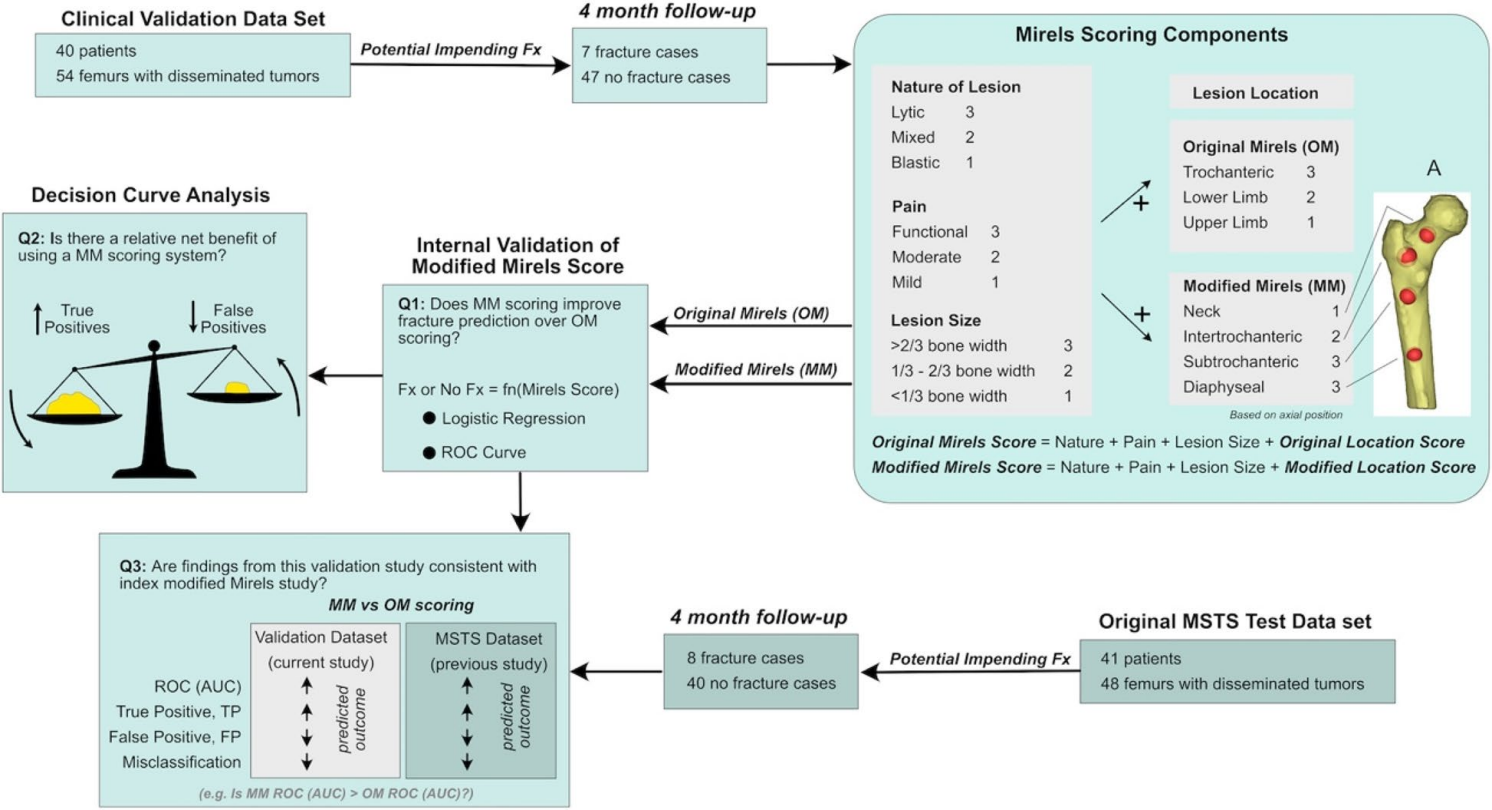
Project workflow. Legend: Workflow for the internal validation of Modified Mirels’ scoring.Figure illustrates patient data collection and approach to assign Original Mirels (OM) and Modified Mirels (MM) scoring. Logistic regression and decision curve analysis were then used to test the quantify the improvement in fracture prediction (Q1 and Q2). Results from validation dataset were then compared to the index MSTS dataset (Q3)

**Fig. 3:**
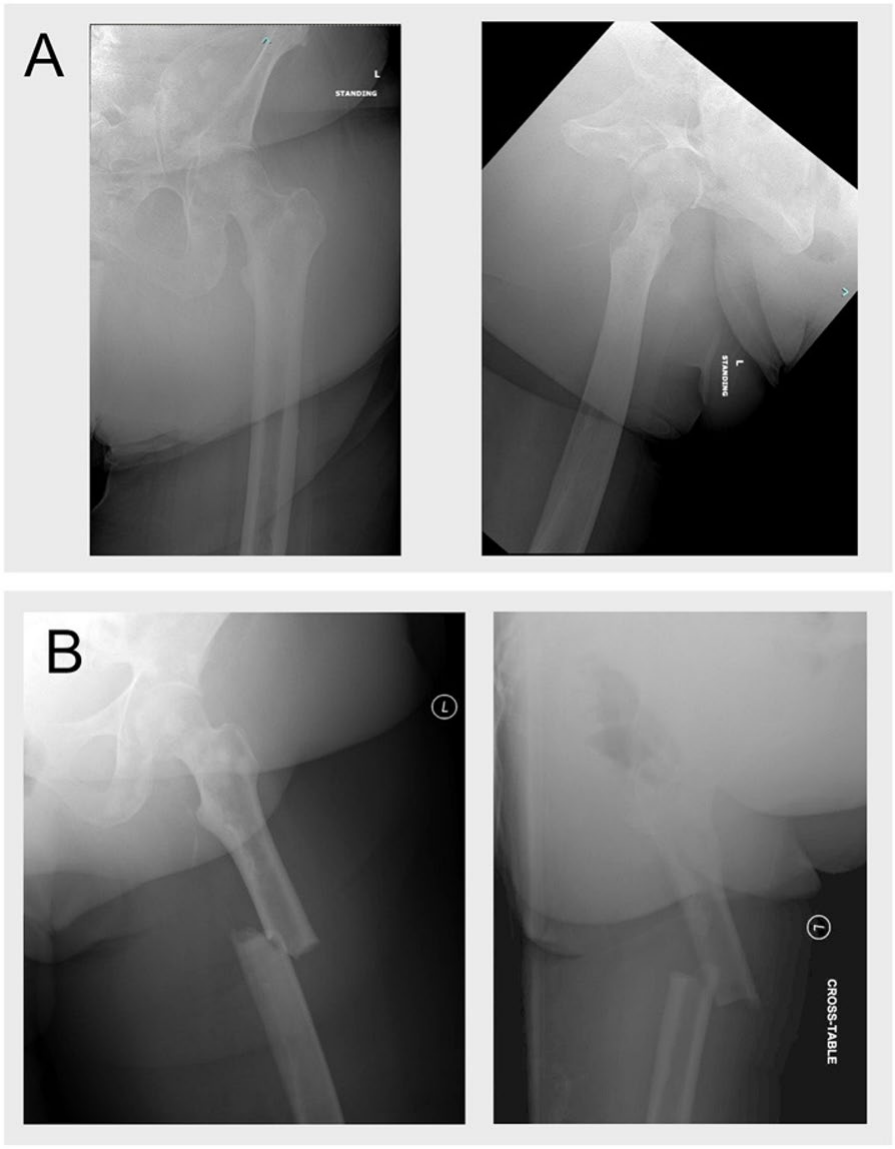
Patient example with fracture. Legend: A female patient presented with metastatic breast cancer involving bilateral proximal femurs as well as lesions in the left femoral diaphysis. Mirels score for the left femoral lesions was rated as 3 for pain, 2 for size, 1 for lesional characteristics. The femur had lesions in the diaphysis and intertrochanteric region resulting in a location score of 3 for Original Mirels and Modified Mirels scoring (**A**). Overall Original and Modified Mirels score of 9 was calculated. The patient had stable pain levels and was not interested in surgery; the decision was made to observe her. The patient then had a ground level fall 2 months later and sustained a pathologic fracture through the diaphyseal lesion requiring cephalomedullary fixation (**B**)

**Fig. 4:**
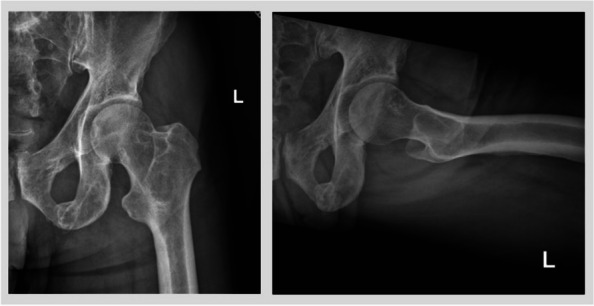
Patient Example without Fracture. Legend: A male patient with known disseminated multiple myeloma presented with a lytic lesion in the intertrochanteric region of the left proximal femur. The original Mirels score at the time of presentation was 9 (1 for pain, 2 for size, 3 for lesional characteristics, and 3 for location). Utilizing the Modified Mirels scoring system with a 2 score for location, the patient would be assigned a score of 8. Due to limited symptoms, he did not undergo prophylactic fixation. The patient remained free of fracture at latest available follow-up which, in this case, exceeded 2 years

Electronic medical records were reviewed to obtain patient data and imaging to ascertain a Mirels’ score at the time of initial evaluation. In most cases, the OM score had been calculated and directly notated in the patient’s record at the time of initial evaluation by the attending physician (senior author). Utilizing patient records and plain radiographs, a MM score was then calculated for all patients retrospectively, but only modifying the location component of the four component OM score. Whereas the OM location score component assigns 1 for upper extremity, 2 for lower extremity, and 3 for peritrochanteric lesions, the MM score assigned scores of 1 for the femoral neck region, 2 for the intertrochanteric region, and 3 for the subtrochanteric and diaphyseal regions (Fig. 2). The femoral neck was defined as the portion of the bone that connects the head with the shaft. The intertrochanteric region was defined as the portion of the femur between the base of the femoral neck and inferior aspect of the lesser trochanter. The subtrochanteric region was defined as within 5 cm of the inferior aspect of the lesser trochanter. The computational modeling approach used in the development of the MM location score is included in the Discussion. Remaining components of the scoring system include pain, lesional characteristics, and lesion size were identical between the two scoring systems. Patients with multifocal lesions were assigned a location score consistent with the highest scoring individual lesion. Example cases are shown in Figs. 3 and 4.

### Demographics

Patient baseline demographic data was similar for the no fracture and fracture groups (Table 1**)**. The demographics of the original MSTS test dataset also matched the validation dataset. The most notable difference between the original MSTS test dataset and the current validation set was the higher (8.2%) original Mirels’ score, although this difference was not statistically significant (*p* = 0.25).

**Table 1:** Patient demographics

	*Validation dataset*				*Original MSTS Test Dataset*			
					*Femurs with no Fx*^a^		*Femurs with Fx*^a^	
	*Patients*	*Femurs with No Fracture*^a^* (Count or Mean (SD))*	*Femurs with Fracture*^b^* (Count or Mean (SD))*	*Fracture vs No Fracture Groups [t-test]*	*Count or Mean (SD)*	*% Difference from validation dataset* *[t-test]*	*Count or Mean (SD)*	*% Difference from validation dataset [t-test]*
Number of Patients/Femurs	40	47	7		40		8	
Sex (F | M)	20 | 20	24 | 23	4 | 3		26 | 14		7 | 1	
Age (yrs)	62.1 (12.4)	60.9 (12.7)	64.7 (11.0)	[0.43]	62. 1 (14.0)	1.9% [0.69]	63.7 (14.9)	1.5% [0.89]
Height (m)	1.67 (0.11)	1.67 (0.11)	1.68 (0.13)	[0.78]	1.64 (0.12)	2.4% [0.29]	1.64 (0.13)	2.4% [0.57]
Weight (kg)	79.8 (22.1)	79.5 (22.8)	88.1 (25.8)	[0.43	77.6 (21.8)	2.3% [0.70]	83.0 (31.9)	5.6% [0.74]
BMI (kg/m^2)	28.2 (6.5)	28.2 (6.4)	31.1 (9.1)	[0.44]	28.8 (7.7)	2.1% [0.68]	30.0 (8.5)	3.5% [0.81]
OM score		8.6 (1.2)	9.7 (1.2)	[0.042]	8.5 (1.04)	1.1% [0.70]	10.5 (1.4)	8.2% [0.25]

*Legend*: Patient demographics for validation and MSTS test dataset. For the validation dataset, 26 patients had unilateral femoral lesions, while 14 had bilateral lesions. Original Mirels’ (OM) score for the Validation and MSTS datasets is also shown. Mean and (standard deviation) values shown. Two-sample t-test *p*-values are reported in [brackets]

^a^Femurs with No Fracture” indicates cases that did not fracture during 4 month follow-up

^b^Femurs with Fracture” represents cases that had a fracture during 4 month follow-up

The most common diseases within the study population were metastatic breast cancer, multiple myeloma, and metastatic lung cancer (Table 2). The intertrochanteric region was the most common lesion location for femurs that did not fracture (Table 3). The subtrochanteric region was the most common lesion location for femurs that fractured, although sample size in these groups/regions was very small. Detailed information for the seven fracture patients is included in Table 4.

**Table 2:** Lesion etiology

*Lesion etiology*	*Patient count* *N (%)*	*Femur count* *No fracture* *N (%)*	*Femur count fractured* *N (%)*
Metastatic breast	10 (25.0)	12 (25.5)	2 (28.6)
Multiple myeloma	12 (30.0)	15 (31.9)	3 (42.9)
Metastatic prostate	5 (12.5)	7 (14.9)	0
Metastatic lung	4 (10.0)	3(6.4)	1 (14.3)
Metastatic renal	3 (7.5)	3 (6.4)	1 (14.3)
Lymphoma	3 (7.5)	4 (8.5)	0
Other (Bladder, SCC Anus, thyroid, uterine)	3 (7.5)	3 (6.4)	0

*Legend*: Patient and femur case distribution based on lesion etiology. Number and percentile (%) of cases

**Table 3:** Lesion location

*Lesion location*	*Femur count – no fracture* *N (%)*	*Femur count—fractured* *N (%)*
Head/neck	8 (17.0)	0 (0)
Intertrochanteric	22 (46.8)	2 (28.6)
Subtrochanteric	3 (6.3)	3 (42.9)
Diaphyseal	12 (25.3)	2 (28.6)
Diffuse	2 (4.2)	0 (0)

*Legend*: Femur case distribution based on lesion location. For instances where there were multiple lesions in a femur, the more distal lesion is listed here. Number and percentile (%) of cases

**Table 4:** Detailed demographics of the 7 fracture cases

*Primary cancer*	*Age (yrs)*	*Sex*	*Lesion location*	*Original Mirels score*	*Modified Mirels score*	*Time to fracture* (months)	*Notes*
*Multiple myeloma*	*60–69*	*M*	*Subtrochanteric*	*10*	*10*	*4*	*“Sneeze” and fall*
*Renal cell*	*80–89*	*M*	*Diaphyseal*	*10*	*11*	*1*	*Fx due to fall before surgery could be done*
*Lung*	*50–59*	*F*	*Subtrochanteric*	*9*	*10*	*1*	*Fall*
*Multiple myeloma*	*50–59*	*M*	*Subtrochanteric*	*11*	*11*	*1.5*	*Climbing into vehicle*
*Breast*	*50–59*	*F*	*Subtrochanteric*	*11*	*10*	*0.5*	*Fx standing from a seated position before surgery could be done*
*Multiple myeloma*	*60–69*	*F*	*Diaphyseal*	*8*	*9*	*1*	*Fall*
*Breast*	*70–79*	*F*	*Diaphyseal*	*9*	*10*	*2*	*Fall*

### Statistical analysis

Descriptive statistics was used to characterize the patient demographics stratified by fracture outcome. While not strictly independent for cases with bilateral femoral lesions, each femur was considered to be independent for statistical analysis.

To determine if MM scoring improved fracture prediction over OM scoring for the validation dataset (*Research Question 1*), logistic regression was used with fracture status as the dependent variable and Mirels’ scoring scheme as the independent variable. The probability of fracture was then determined as a function of Mirels’ score. Receiver operator characteristic (ROC) curves were generated to evaluate the interaction between sensitivity and specificity for OM and MM scoring. The area under the ROC curve (AUC) was calculated as an overall measure of fracture prediction performance. JMP Pro 17 (SAS, Cary, NC) was used for all statistical analyses.

### Decision Curve Analysis (DCA)

To determine if there was a *net benefit* of using MM scoring over OM scoring, decision curve analysis (DCA) was used (*Research Question 2*). DCA is an approach to assess the clinical value of a diagnostic test in the context of acceptable levels of true positives (*TP*, correctly identifying femurs that will fracture) and false positive (*FP*, incorrectly predicting pathologic fracture) predictions [27, 29]. A simple type of decision curve calculates the net benefit (*NB*) of a particular scoring system by weighing the benefits (*TP*) and harms (*FP*):

1 $$NB=\frac{TP}{N}-\frac{FP}{N}\left(\frac{{p}_{t}}{1-{p}_{t}}\right)$$

where *p*_*t*_ is threshold probability of fracture for the number of subjects (femurs) in the study (N).

The threshold probability of fracture is a variable from 0 to 100% and is used to weight the importance of *TP* and *FP* on the same scale. For example, a threshold probability of 0.1 (10%) would weigh the benefit of finding one true positive the same as the cost of treating nine false positives (NB = TP – $\frac{1}{9}$ FP). A *p*_*t*_ of 50% would represent a situation where finding a true positive was of the same importance as allowing a false positive. For this study, OM and MM scoring were plotted on the same net benefit graph as a function of threshold probability. This allowed for a head-to-head comparison of the effectiveness of the two scoring systems over a range of relevant threshold probabilities. The reduction in false positives for MM scoring relative to OM scoring can also be calculated using:

2 $$FP Reduction=\frac{{NB({p}_{t})}_{MM}-{NB({p}_{t})}_{OM}}{({p}_{t}/\left(1-{p}_{t}\right))}x 100 for {p}_{t}>0$$

This equation was used to document to relative net benefit of using MM over OM scoring as a function of acceptable threshold probability of fracture.

### Comparison with index evaluation of OM and MM scoring

The improvement in the ability to correctly identify femurs at risk of fracture using MM scoring for the current dataset was compared to results reported [1] from the original assessment of the MM scoring system using ROC and AUC measures. Finally, a standard application of Mirels’ scoring (using a Mirels’ score of 9 or greater as an indicator of impending pathologic fracture) was used to demonstrate the accuracy of the OM and MM scoring predictions was evaluated for the index and validation study (*Research Question 3*).

## Results

### Research question #1: does MM scoring improve fracture prediction over OM scoring?

**Fig. 5:**
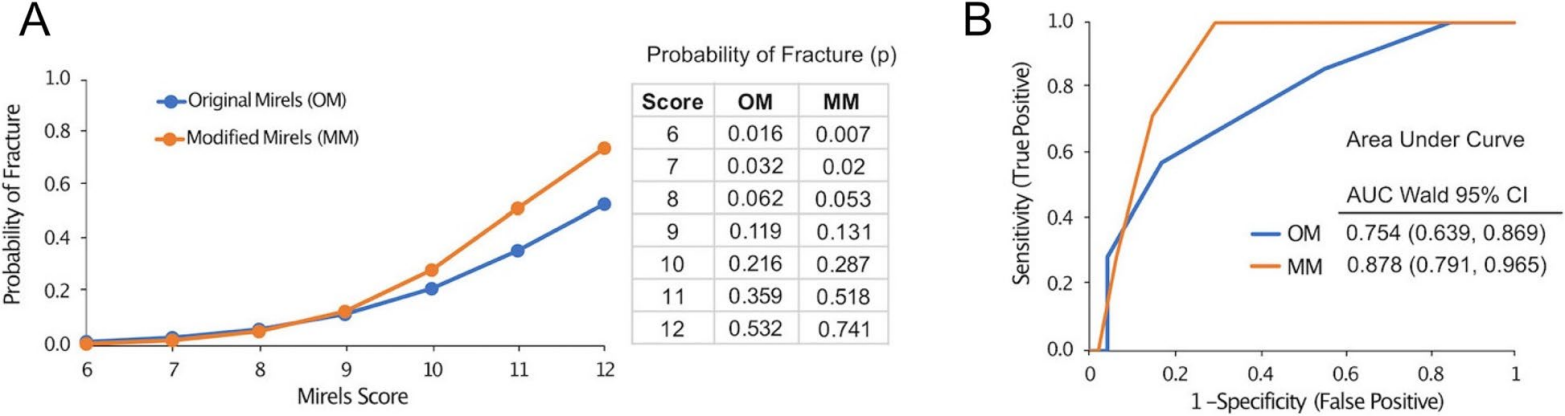
Logistic regression results. Legend: Probability of fracture from logistic regression (**A**) and Receiver Operator Characteristic Curves (ROC) (**B**) for Original Mirels’ (OM) and Modified Mirels’ (MM) scores in patients with potential impending pathologic fractures

The use of the Modified Mirels’ (MM) scoring improved fracture prediction over Original Mirels’ (OM) scoring for patients observed over a 4 month follow up based on logistic regression. The probability of fracture for a Mirels’ score of 9 or less was similar for the two scoring methods, but for higher Mirels’ scores (> 9) the probability of fracture was greater for the MM scoring (Fig. 5A). For a patient with a Mirels’ score of 11, the probability of fracture was predicted as 36% using OM scoring, but increases to 52% for MM scoring, thus increasing confidence that a fracture would occur. The corresponding Receiver Operator Characteristic (ROC) curves plotting sensitivity and specificity shows that the MM scoring area under the curve (AUC) (0.878) was greater than that of the OM scoring (0.754) (Fig. 5B).

### Research question #2: what is the net benefit of using MM scoring over OM scoring?

**Fig. 6:**
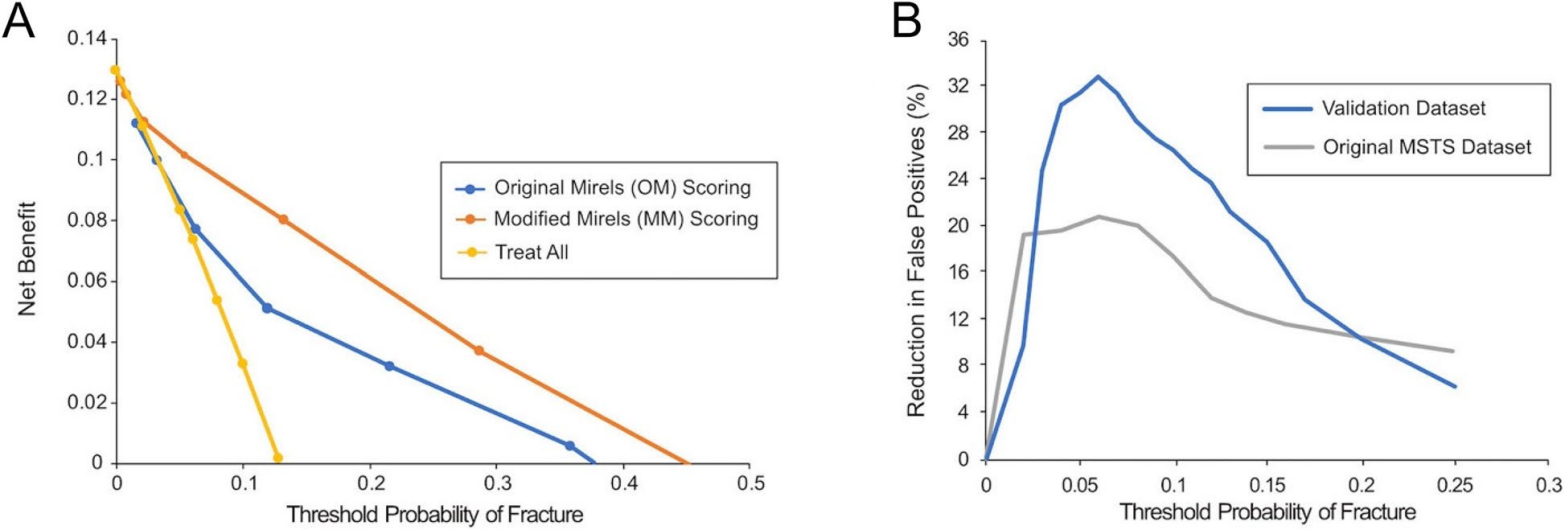
Decision curve analysis. Legend: Decision curve analysis showed net benefit of both the Original and Modified Mirels’ Scores over a range of threshold probabilities (**A**). The reduction in false positives (**B**) for the current validation dataset and the original MSTS dataset are shown

Decision curve analysis (DCA) showed that there was a net benefit (NB) using the MM score over the OM scoring for a full range of fracture threshold probabilities (Fig. 6A). Note that a threshold probability of 0 represents a theoretical scenario where no fractures would be allowed, and in this case, all patients would be prophylactically stabilized. The ‘treat all’ line represents this theoretical scenario, and at a threshold probability of zero, represents the prevalence of fractures (7 fractures in 54 femurs, NB = 13%) in the validation dataset.

For fracture threshold probabilities greater than ~ 3%, there was a NB using MM scoring over ‘treat all’. For OM scoring, there was a NB over ‘treat all’ when threshold probability was greater than ~ 6%. Note that the 6% probability of fracture corresponds to an OM score of 8 as shown in Fig. 6A; an OM score of 8 is considered to be a borderline score for impending fracture [18]. For the current study population, this shows that OM scoring has utility in estimating fracture risk above ‘treat all’ for OM scores greater than 8, but is inferior to the MM scoring over a full range of fracture probabilities.

The reduction of false positives using MM scoring over OM scoring (Fig. 6B) was calculated as a function of fracture threshold probability using Eq. 2. The graph can be clinically interpreted as follows: If a surgeon were willing to prophylactically stabilize 10 patients in order to prevent one fracture, use of the MM scoring in place of an OM scoring would reduce the number of false positives fracture predictions by about 25%. It is important to note that the reduction in false positives occurs without a loss of sensitivity (detection of true positives).

### Research question #3: are validation study results consistent with original MM study?

Fracture prevalence was similar for current internal validation dataset when compared to the MSTS dataset (Table 5). The AUC was greater for the MM scoring when compared to OM scoring in both validation and MSTS datasets. Using a Mirels’ score of 9 and above (9 +) as a cut-off threshold to describe impending pathological fracture, a reduction in false positive rate for MM score was found for the validation and MSTS datasets. Overall, misclassification of fracture prediction was substantially reduced for MM scoring for the internal validation and original MSTS datasets. The reduction in false positives reached a greater magnitude (Table 5) for the validation dataset when compared to the original MSTS dataset. This is likely due to difference in ROC curve AUC for MM scoring versus OM scoring (∆ AUC = 0.878 – 0.754 = 0.124) in the validation dataset when compared to differences in AUC for the original MSTS dataset (∆ AUC = 0.941 – 0.853 = 0.088).

**Table 5:** Classification characteristics

	**Internal validation dataset**		**Original MSTS dataset**	
Patient image source	Radiograph		CT Scan	
Number of cases	7 Fracture | 47 No Fracture		8 Fracture | 40 No Fracture	
Fracture prevalence (%)	12.9		16.7	
	OM Scoring	MM Scoring	OM Scoring	MM Scoring
ROC AUC	0.754 (0.639, 0.869)	0.878 (0.791, 0.965)	0.853 (0.777, 0.929)	0.941 (0.891, 0.991)
True positive rate (%)	85.7 (48.7,97.4)	100 (64.5, 100)	100 (67.5, 100)	87.5 (67.6, 100)
False positive rate (%)	55.4 (41.3, 68.6)	29.8 (18.7, 44.0)	55.0 (39.1, 69.3)	37.5 (22.2, 50.5)
PPV (%)	18.7 (8.9, 35.3)	33.3 (17.2, 54.6)	26.7 (14.2, 44.4)	34.7 (19.7, 57.0)
NPV (%)	95.4 (78.2, 99.2)	100 (89.5, 100)	100 (82.4, 100)	100 (87.1, 100)
Misclassification (%)	50.0	25.9	45.8	31.2

*Legend*: Classification characteristics of impending pathological fracture using Mirels’ score of 9 or greater (9 +) as the cut-off threshold for current internal validation dataset and initial MSTS study test dataset. Results are shown for Original Mirels’ (OM) scoring and Modified Mirels’ (MM) scoring. Mean and (95% Confidence Interval) values are shown for each parameter

## Discussion

### Overall findings

Use of Modified Mirels’ (MM) scoring was found to improve fracture prediction over Original Mirels’ (OM) scoring when tested on an internal validation set of patients with disseminated metastatic lesions to the proximal femur. There was greater ROC area under the curve (AUC) for MM scoring and there was a net benefit to using MM over OM scoring over the full range of threshold probabilities of fracture. The superiority of MM over OM found with the validation dataset was consistent with the results found using the MSTS test dataset. In practice, using a MM score of 9 or higher to indicate an impending pathological fracture showed a substantial reduction in the false positive rate, with little change in the true positive rate, and a nearly 50% reduction in the misclassification of fracture prediction when compared to OM scoring.

### What is an appropriate cutoff to identify patients at risk of pathologic fracture?

Based on Mirels [18] historic development of the scoring system of impending pathological fractures, Mirels concluded that lesions with a score of 7 or lower would not need prophylactic stabilization, while lesions with a score of 8 or higher would require stabilization to prevent an impending pathologic fracture. Comparing the *Mirels 1989 dataset* with the current *combined* test and internal validation set (Table 6), we see a large discrepancy between the fracture probabilities predicted from Mirels 1989 dataset and current combined dataset. An OM score of 8 for the Mirels 1989 dataset resulted in a calculated 15% probability of fracture using logistic regression. If an OM score of 8 was used in the current study as the cutoff, the fracture probability would be only 4%, and more importantly, the number of false positives would grow substantially using this cutoff. Recalibrating the OM score of 8 from Mirels 1989 (15% fracture probability) would correspond roughly to an OM 9 score (11% fracture probability) and MM 9 score (12.7% fracture probability) for the current study.

**Table 6:** Historic Mirels 1989 study

*Dataset*	*Mirels 1989*	*Current COMBINED Test & Internal Validation Dataset*	
Number of patients	38	80	
Fracture prevalence	35%	14.8%	
Follow-up period	6-month w/radiation therapy	4-month	
Cancer etiology	Breast 64%, Myeloma 14%, Prostate 7.6%, Lung 6.4%	Breast 25%, Myeloma 30%, Prostate 12.5%, Lung 10%	
Lesion locations	Peritrochanteric 58%, Upper extremity 22%, lower extremity 20%	Proximal Femur 100%	
	***Probability of Fracture (p)***		
Mirels’ score	OM	OM	MM
4	0	0	0
5	0	0	0
6	0	0	0
7	0.04	0.015	0.01
8	0.15	0.042	0.037
9	0.33	0.11	0.127
10	0.72	0.264	0.36
11	0.96	0.51	0.78
12	1.0	0.75	0.89

*Legend*: Comparing the patient characteristics and fracture probabilities for the historic Mirels 1989 study and current study with COMBINED test and internal validation datasets

It should be noted that the Mirels 1989 and current datasets are different in terms of fracture prevalence and lesion locations, with the current study restricted to the proximal femur (Table 6). The majority of the lesions were from patients with breast cancer in the Mirels 1989 dataset, while this etiology accounted for only 25% of the patients in the current study. A substantial fraction (35%) of fractures occurred in non-weight-bearing regions in the Mirels 1989 dataset and would have a location score of 1, which would lower the OM score when compared to the current study. It is also likely that cancer treatment modalities have changed substantially over the 25-year period between these two studies. All of these factors could contribute to the differences found in the calculated fracture probabilities for the Mirels 1989 and current dataset.

The results from the current COMBINED Test & Internal Validation Dataset support using a MM score of 9 as a cutoff for classifying a patient at risk of pathologic fracture. Based upon our findings, a change in the definition of impending pathologic fracture to a MM score of 10 or higher was considered, as it would reduce the false positive rate even further, from 33% at a MM threshold score of 9 to just 10.4% for a MM score of 10. However, doing so would also negatively impact sensitivity, reducing the 100% sensitivity with MM score of 9 as threshold to 73% for MM score of 10. Hence, the authors recommend continued use of a score of 9 in the MM as the definition of an impending pathologic fracture. Expanding this current dataset with external validation is needed to fully endorse a specific cutoff.

### Study limitations

A limitation of this internal validation study was the limited number of fracture cases (*n* = 7) relative to the number of no fracture cases (*n* = 47). Ideally from an experimental perspective, the number of fracture cases that occurred after the initial evaluation would be larger, but these were uncommon occurrences in practice. This is likely due to the fact that most patients assigned a high Mirels’ score for metastatic lesions in the proximal femur would have been prophylactically stabilized. Of course, in those cases, it could not be determined whether fracture would occur because of interference with the natural history. If the validation dataset were combined with the original MSTS dataset (15 fractures / 87 no fracture), we found that the MM AUC (0.913) was significantly greater than the OM AUC (0.807) (*p* = 0.027, MedCalc Software Ltd, Ostend, Belgium). This further supports the conclusion that the MM scoring system is more accurate in assessing fracture risk for patients with metastatic lesions in the proximal femur.

A second limitation of the study was that the location score assignment was based on assumptions made in a previous computational study [1] to calculate the loss of femoral strength for normal femurs with idealized lytic lesions (example of lesion locations shown in Fig. 2). The lesions were simulated through removal of bone in an idealized spherical shape at specific locations. The effect of lesion location (axial position) on loss of femur strength was then determined. A Modified Mirels’ location score was assigned a 1 (> 75% normal femur strength), 2 (50–75% normal femur strength), or 3 (< 50% normal femur strength). The shape of the lesion and variable lesion properties (lytic, mixed, blastic) were not considered in the modeling. Further, the modeling was not patient-specific. Finite element modeling [7–9, 12, 17, 22, 24, 26] or structural rigidity analysis [4, 6, 19, 20] based directly on patient CT scans would likely produce more accurate assessments of loss of femur strength due to the metastatic lesions. Access to these advanced analysis tools remain limited to specialized centers, but if available, could serve as another independent tool to estimate fracture risk.

A third limitation was that the imaging approaches for the MSTS dataset (density calibrated CT scans) and validation dataset (planar radiographs) were different. While both datasets produced similar conclusions regarding validity of MM scoring, it is not known if the less granular nature of planar imaging (when compared to 3D CT imaging) alters the assignment of location and size scores. One benefit of using radiographic based imaging is that it is more commonly used as a first screening tool and thus there would likely be the potential for collection of a greater number of clinical cases when compared to CT imaging.

### Limitations of Mirels scoring

The motivation behind the Modified Mirels scoring project was to investigate whether a simple change is the scoring of the location component (informed by computational modeling studies) would improve fracture prediction. All other aspects of Mirels scoring remained the same and the approach of summing the scoring components was preserved. A limitation of this summed-scoring approach is that all parameters are weighted equally (eg a large lesion (3 score) has the same weight as functional pain (3 score)). In addition, there is no accounting for interactions or correlations between components (eg a very small lesion (1) in the diaphysis (3) has the same sub-score (4) as a very large lesion (3) in the femoral neck (1)). Post-hoc multivariate analysis of correlations between the four Mirels scoring components (Table 7) shows that there was negligible correlation between most scoring pairs, except there was a weak positive correlation between MM location score and type (*p* = 0.0074).

**Table 7:** Pairwise correlations between the four Mirels scoring parameters

Variable	By variable	Correlation	*P*-value
Size	Pain	-0.035	0.799
Type	Pain	-0.254	0.063
Type	Size	-0.036	0.795
MM location	Pain	-0.138	0.321
MM location	Size	0.135	0.329
MM location	Type	0.361	**0.0074**

Scoring of pain may be more subjective compared to the other three radiographic-based measures. Other patient factors such as rate of tumor progression, risk of falling for the patient, and confounding risk of osteoporotic fracture of the femur are not included. Improved prediction of fracture risk which included these lesion characteristics and patient factors could be achieved using machine learning (AI) approaches as has been performed recently for longevity prediction for patients with metastatic lesions (PathFx) [11]. Machine learning approaches would have the potential to find high-risk combinations of patient factors that are not revealed using a simple, single-numbered scoring approach. But this would require a large training and validation datasets in order to develop a robust and accurate fracture prediction tool.

### Decision curves analysis and net benefit

Logistic regression (with generation of a receiver operator curve (ROC)) is a common statistical approach to assess a binary classifier (fracture or no fracture) or prediction tool. Here, we showed that both OM and MM scoring have utility with AUC values of between 0.75 and 0.94, which is much greater than a random prediction (AUC = 0.5). An AUC of 1.0 would indicate correct prediction for all fracture and no fracture cases. However, the AUC does not describe the shape of the ROC curve, and two prediction tools could have the same AUC, but different utility in practice. This is because the AUC measure does not describe the combinations of true and false positives as a function of clinically relevant fracture threshold probabilities. Decision curve analysis (DCA) was developed [10, 27, 28] to graphically present this information and to allow comparison between different scoring tools.

The net benefit calculation weighs the benefit of identifying true positives (femurs that would fracture) and costs of false positive (femurs incorrectly predicted to fracture) with threshold probability as the weighting factor. MM scoring has a higher net benefit for all threshold probabilities > 0.03 (3%). Thus, MM scoring should be used (over OM) irrespective of what one would consider to be an appropriate threshold probability of fracture (Fig. 6A). Plotting the reduction in false positives with use of MM when compared to OM scoring allows for easier clinical interpretation (Fig. 6B). For example, if a surgeon were willing to treat 20 femurs to prevent one fracture (0.05 threshold probability) then MM has much greater utility over OM scoring with a ~ 30% reduction in false positives. This occurs without any loss of identifying true positives.

## Conclusion

In summary, this study validates MM scoring as an improvement over OM scoring for patients with proximal femoral MBD. The improvement in fracture prediction demonstrated in the present study mirrored the results of our index study during which the Modified Mirels’ system was developed. Our index study showed that through simple modification of the location component of Mirels’ scoring system, there was a net benefit to using a Modified Mirels’ scoring and that implementation could reduce the number of false positives (patients predicted to fracture, but did not). Both our index study and the current study correlate with previously reported biomechanical data demonstrating higher strain patterns across the diaphyseal and subtrochanteric regions of the femur compared to intertrochanteric and neck [16, 23].

Although use of the MM is supported by this study, further external validation is encouraged before widespread acceptance. The relevance of a purely x-ray based prediction system such as the MM can be questioned in the context of development of more sophisticated techniques such as CTRA (CT-based Rigidity Analysis) and FEA (Finite Element Analysis). However, those analysis techniques remain inaccessible to the vast majority of orthopedic surgeons and almost universally require time delays that are not practical to clinical decision-making. Use of CT without those tools may be favored by some, but without objective verification of it’s use, CT interpretation in this context remains subjective. Hence, any improvement in the original scoring system such as the MM should be considered an advance in the “front-line” care of these patients on a day-to-day basis.

## Data Availability

Deidentified datasets used and analyzed during the current study are available as supplemental files on request by contacting the corresponding author.

## References

[1] Amendola RL, Miller MA, Kaupp SM, Cleary RJ, Damron TA, Mann KA. Modification to Mirels scoring system location component improves fracture prediction for metastatic disease of the proximal femur. BMC Musculoskelet Disord. 2023;24:65. 36694156 10.1186/s12891-023-06182-7 PMC9872372

[2] Anez-Bustillos L, Derikx LC, Verdonschot N, Calderon N, Zurakowski D, Snyder BD, Nazarian A, Tanck E. Finite element analysis and CT-based structural rigidity analysis to assess failure load in bones with simulated lytic defects. Bone. 2014;58:160–7. 24145305 10.1016/j.bone.2013.10.009 PMC3908856

[3] Crenn V, Carlier C, Gouin F, Sailhan F, Bonnevialle P, members of the So FCOT. High rate of fracture in long-bone metastasis: Proposal for an improved Mirels predictive score. Orthop Traumatol Surg Res. 2020;106:1005–11. 32782175 10.1016/j.otsr.2020.03.034

[4] Damron TA, Brown C, Entezari V, Nazarian A, Snyder BD, Hipp JA, Calderon N, Terek RM, Cheng EY, Aboulafia AJ, Anderson ME, Gebhardt MC. CT-based structural rigidity analysis improves specificity over Mirels scoring for fracture prediciton. In: Transactions of the Orthopaedic Research Society. 2012; Abstract 1485.

[5] Damron TA, Mann KA. Fracture risk assessment and clinical decision making for patients with metastatic bone disease. J Orthop Res. 2020;38:1175–90. 32162711 10.1002/jor.24660 PMC7225068

[6] Damron TA, Nazarian A, Entezari V, Brown C, Grant W, Calderon N, Zurakowski D, Terek RM, Anderson ME, Cheng EY, Aboulafia AJ, Gebhardt MC, Snyder BD. CT-based structural rigidity analysis is more accurate than Mirels scoring for fracture prediction in metastatic femoral lesions. Clin Orthop Relat Res. 2016;474:643–51. 26169800 10.1007/s11999-015-4453-0 PMC4746194

[7] Eggermont F, Derikx LC, Verdonschot N, van der Geest ICM, de Jong MAA, Snyers A, van der Linden YM, Tanck E. Can patient-specific finite element models better predict fractures in metastatic bone disease than experienced clinicians?: Towards computational modelling in daily clinical practice. Bone Joint Res. 2018;7:430–9. 30034797 10.1302/2046-3758.76.BJR-2017-0325.R2 PMC6035356

[8] Eggermont F, van der Linden Y, Verdonschot N, Dierselhuis E, Ligthert S, Bitter T, Westhoff P, Tanck E. A patient-specific fracture risk assessment tool for femoral bone metastases: using the Bone Strength (BOS) score in clinical practice. Cancers (Basel). 2022;14:5904. 36497388 10.3390/cancers14235904 PMC9740241

[9] Eggermont F, van der Wal G, Westhoff P, Laar A, de Jong M, Rozema T, Kroon HM, Ayu O, Derikx L, Dijkstra S, Verdonschot N, van der Linden Y, Tanck E. Patient-specific finite element computer models improve fracture risk assessments in cancer patients with femoral bone metastases compared to clinical guidelines. Bone. 2020;130:115101. 31655223 10.1016/j.bone.2019.115101

[10] Fitzgerald M, Saville BR, Lewis RJ. Decision curve analysis. JAMA. 2015;313:409–10. 25626037 10.1001/jama.2015.37

[11] Forsberg JA, Wedin R, Boland PJ, Healey JH. Can we estimate short- and intermediate-term survival in patients undergoing surgery for metastatic bone disease? Clin Orthop Relat Res. 2017;475:1252–61. 27909972 10.1007/s11999-016-5187-3 PMC5339146

[12] Goodheart JR, Cleary RJ, Damron TA, Mann KA. Simulating activities of daily living with finite element analysis improves fracture prediction for patients with metastatic femoral lesions. J Orthop Res. 2015;33:1226–34. 25761000 10.1002/jor.22887

[13] Hoban KA, Downie S, Adamson DJA, MacLean JG, Cool P, Jariwala AC. Mirels’ score for upper limb metastatic lesions: do we need a different cutoff for recommending prophylactic fixation? JSES Int. 2022;6:675–81. 35813136 10.1016/j.jseint.2022.03.006 PMC9264023

[14] Howard EL, Shepherd KL, Cribb G, Cool P. The validity of the Mirels score for predicting impending pathological fractures of the lower limb. Bone Joint J. 2018;100-B:1100–5. 30062934 10.1302/0301-620X.100B8.BJJ-2018-0300.R1

[15] Johnson JE, Goetz JE, Brouillette MJ, Miller BJ. Finite element analysis potentially identifies nonessential prophylactic stabilization in femurs with metastatic disease. Proc Inst Mech Eng H. 2022;236:1297–308. 35787214 10.1177/09544119221109740

[16] Kersh ME, Martelli S, Zebaze R, Seeman E, Pandy MG. Mechanical loading of the femoral neck in human locomotion. J Bone Miner Res. 2018;33:1999–2006. 29920773 10.1002/jbmr.3529

[17] Keyak JH, Kaneko TS, Rossi SA, Pejcic MR, Tehranzadeh J, Skinner HB. Predicting the strength of femoral shafts with and without metastatic lesions. Clin Orthop Relat Res. 2005;439:161–70. 16205155 10.1097/01.blo.0000174736.50964.3b

[18] Mirels H. Metastatic disease in long bones. A proposed scoring system for diagnosing impending pathologic fractures. Clin Orthop Relat Res. 1989;249:256–64. 2684463

[19] Nazarian A, Entezari V, Villa-Camacho JC, Zurakowski D, Katz JN, Hochman M, Baldini EH, Vartanians V, Rosen MP, Gebhardt MC, Terek RM, Damron TA, Yaszemski MJ, Snyder BD. Does CT-based rigidity analysis influence clinical decision-making in simulations of metastatic bone disease? Clin Orthop Relat Res. 2016;474:652–9. 26022114 10.1007/s11999-015-4371-1 PMC4746188

[20] Oftadeh R, Karimi Z, Villa-Camacho J, Tanck E, Verdonschot N, Goebel R, Snyder BD, Hashemi HN, Vaziri A, Nazarian A. curved beam computed tomography based structural rigidity analysis of bones with simulated lytic defect: a comparative study with finite element analysis. Sci Rep. 2016;6:32397. 27585495 10.1038/srep32397 PMC5009360

[21] Riaz S, Bashir H, Niazi IK, Butt S, Qamar F. 99mTc MDP SPECT-CT-based modified Mirels classification for evaluation of risk of fracture in skeletal metastasis: a pilot study. Clin Nucl Med. 2018;43:e180–3. 29561523 10.1097/RLU.0000000000002057

[22] Sas A, Ohs N, Tanck E, van Lenthe GH. Nonlinear voxel-based finite element model for strength assessment of healthy and metastatic proximal femurs. Bone Rep. 2020;12:100263. 32322609 10.1016/j.bonr.2020.100263 PMC7163060

[23] Simoes JA, Vaz MA, Blatcher S, Taylor M. Influence of head constraint and muscle forces on the strain distribution within the intact femur. Med Eng Phys. 2000;22:453–9. 11165142 10.1016/S1350-4533(00)00056-4

[24] Sternheim A, Traub F, Trabelsi N, Dadia S, Gortzak Y, Snir N, Gorfine M, Yosibash Z. When and where do patients with bone metastases actually break their femurs? Bone Joint J. 2020;102-B:638–45. 32349590 10.1302/0301-620X.102B5.BJJ-2019-1328.R2

[25] Toci GR, Bressner JA, Morris CD, Fayad L, Levin AS. Can a novel scoring system improve on the Mirels score in predicting the fracture risk in patients with multiple myeloma? Clin Orthop Relat Res. 2021;479:521–30. 32420721 10.1097/CORR.0000000000001303 PMC7899603

[26] van der Wal C, Eggermont F, Fiocco M, Kroon HM, Ayu O, Slot A, Snyers A, Rozema T, Verdonschot NJJ, Dijkstra PDS, Tanck E, van der Linden YM. Axial cortical involvement of metastatic lesions to identify impending femoral fractures; a clinical validation study. Radiother Oncol. 2020;144:59–64. 31733489 10.1016/j.radonc.2019.10.007

[27] Vickers AJ, Elkin EB. Decision curve analysis: a novel method for evaluating prediction models. Med Decis Making. 2006;26:565–74. 17099194 10.1177/0272989X06295361 PMC2577036

[28] Vickers AJ, Holland F. Decision curve analysis to evaluate the clinical benefit of prediction models. Spine J. 2021;21:1643–8. 33676020 10.1016/j.spinee.2021.02.024 PMC8413398

[29] Vickers AJ, Van Calster B, Steyerberg EW. Net benefit approaches to the evaluation of prediction models, molecular markers, and diagnostic tests. BMJ. 2016;352:i6. 26810254 10.1136/bmj.i6 PMC4724785

